# A pilot feasibility study of *Exercising Together*© during radiation therapy for prostate cancer: a dyadic approach for patients and spouses

**DOI:** 10.1186/s40814-021-00952-7

**Published:** 2021-12-08

**Authors:** Kerri M. Winters-Stone, Karen S. Lyons, Tomasz M. Beer, Meghan B. Skiba, Arthur Hung

**Affiliations:** 1 Knight Cancer Institute, Oregon Health and Science University, Portland, OR USA; 2 Connell School of Nursing, Boston College, Boston, MA USA

**Keywords:** Prostate cancer, Exercise, Physical function, Mental health, Relationship, Dyad, Physical activity, Caregiver

## Abstract

**Introduction:**

Prostate cancer can negatively impact the health of patients and their spouse, particularly early on in the cancer trajectory.

**Purpose:**

To determine the feasibility and acceptability of dyadic exercises during radiation therapy and preliminary efficacy on physical, mental, and relational outcomes for men and their spouses. *Exercising Together©*, originally designed as a 6-month dyadic resistance training program for couples post-treatment, was adapted for the radiation setting.

**Methods:**

We conducted a single-group pilot feasibility study of *Exercising Together©* in men scheduled for radiation therapy for prostate cancer and their spouse*.* Couples attended supervised exercise sessions thrice weekly throughout radiation treatment and were followed up 8 weeks later. Primary outcomes were feasibility and acceptability with secondary outcomes of changes in physical (physical functioning (short physical performance battery (sPPB)), gait speed (m/s), functional capacity (400-m walk (min), physical activity (min/week)), mental (depressive symptoms (CES-D), and anxiety (SCL-90 ANX)), and relationship (Dyadic Coping, Role Overload, and Physical Intimacy Behavior Scales) health outcomes for each partner. Participants completed an evaluation post-intervention.

**Results:**

Ten couples enrolled and 8 completed the intervention, attending 83% of scheduled sessions. Couple satisfaction with the intervention was high (patients: mean difference (MD) = 9.4 ± 1.9 and spouses: MD = 10.0 ± 0.0, on a 1–10 scale). At post-intervention, gait speed (MD = 0.1; 95%CI: 0.1, 0.2; *p* = 0.003; *d* = 0.94) and functional capacity (MD = −0.6; 95%CI: −0.9, 0.3; *p* = 0.002; *d* = −0.42) improved in patients and sPPB in spouses (MD = 1.3; 95%CI: 0.3, 2.2; *p* = 0.02; *d* = 0.71). Total physical activity increased non-significantly for patients and significantly for spouses at post-intervention and decreased at follow-up (MD = 179.6; 95%CI: 55.4, 303.7; *p* = 0.01; *d* = 1.35 and MD = −139.9; 95%CI: −266.5, 13.3; *p* = 0.03; *d*=1.06). Among patients, anxiety and active engagement significantly improved post-intervention (MD = −2.3; 95%CI: −3.8, 0.7; *p* = 0.01; *d* = −0.43 and MD = 2.5; 95%CI: 0.7, 4.3; *p* = 0.01; *d* = 0.98, respectively). There were modest effects on other physical, mental, and relationship health domains in patients and spouses.

**Conclusion:**

A modified version of *Exercising Together©* is a feasible and acceptable program during radiation therapy for prostate cancer and shows preliminary evidence for improvements on physical, mental, and relational health in both patient and spouse. A larger, fully powered randomized controlled trial is warranted and could help shift the landscape toward dyadically targeted interventions.

**Trial registration:**

This study was registered on ClinicalTrials.gov on February 18th, 2018 (NCT03418025).

## Key messages regarding feasibility

- What uncertainties existed regarding the feasibility?

The modified version of the *Exercising Together©* program is significantly shorter than the original version and was also administered at the beginning of radiation treatment instead of after treatment completion. Thus, it was not known if couples would enroll in a facility-based program at the start of therapy, if they would attend a sufficient number of classes, nor if they would complete the exercise program and assessments.

- What are the key feasibility findings?

Couples who are newly adjusting to the new diagnosis of prostate cancer are interested and willing to participate in a facility-based, thrice weekly exercise program together, and are also able to complete both performance-based and self-report assessments. Follow-up assessments, however, might only be possible using self-report as couples may leave the vicinity of their treatment facility after therapy ends.

- What are the implications of the feasibility findings for the design of the main study?

A dyadic resistance exercise program is feasible during radiation cancer treatment and should be more rigorously tested; however, when and how the program and assessments are delivered should be tailored to the logistics surrounding couple availability.

## Background

Two million prostate cancer survivors are alive in the USA and this number will double in just a few decades [1]. Health-related quality of life is lower in prostate cancer patients with increased symptom presence and severity [2–4], men who receive treatments in addition to prostatectomy [2, 5], and men with advanced disease [6]. Cancer survivors are also twice as likely to report a limitation in activities of daily living and/or an inability to work due to poor health than the general population [7–10]. Most cancer survivors are married when diagnosed and cancer will also threaten the physical and mental health of their aging spouse and the quality of their marital relationship. Within couples, the cancer experience can contribute to adverse health outcomes. Spouse care partners also experience significant health declines and are at greater risk for mortality and mobility limitations than other family care partners [11–16]. Both survivor and spouse experience high levels of psychological distress and depression from the cancer experience [17–22], including depressive symptoms and anxiety [23, 24]. Such high levels of psychological distress can have long-term negative consequences for both partners and compromise the ability of the spouse care partner to provide quality care [25]. Cancer also strains the marriage by hampering communication [26, 27] and interfering with sex [28, 29] which in turn erodes the emotional and physical intimacy that protects couples from the consequences of illness [30–36]. Yet, few interventions focus on the couple as a unit.

The Theory of Dyadic Illness Management [37], a new conceptual framework, proposes that illness management is a dyadic phenomenon because partners experience and manage an illness, such as cancer, together. This theory focuses extensively on the dyad as an interdependent team with a goal to optimize the health of both members of the dyad. The model views the illness experience as an interpersonal process, with dyadic (physical and mental) health the result of how the couple appraises and manages the illness as a unit (i.e., collaboration, communication, and supportive behaviors). Specifically, couples that engage in dyadic health management strategies towards a common goal (e.g., better physical health) are more likely to experience more positive health outcomes. An explicit strength of the model is that it moves beyond an individualistic approach to focus on the transactional nature of how patients and care partners influence one another and acknowledges the important roles that collaborative management strategies have for both members. Couple-based interventions, in the broader chronic illness context, are more effective than individual-based interventions for improving individual and couple psychosocial and relational outcomes [38]; however, few, if any dyadic interventions have addressed physical, mental, and relational health all at once.

Exercise could improve the physical and mental health of both patients and spouses. New evidence-based recommendations for cancer survivors indicate that resistance training can reduce fatigue, improve physical functioning, and quality of life [39]. Resistance training may be the ideal exercise for aging cancer survivors and their spouses because this exercise mode is the most widely recommended form of exercise to combat age-related declines in muscle mass and physical function and has additional health benefits including better mood [40–42] and decreased mortality. Even though the role of exercise to prevent and manage illness is increasingly known, rates of inactivity among older adults are higher than any other age group. Many men are inactive when they begin cancer treatment and among physically active cancer patients, physical activity typically decreases during cancer treatment and rarely recovers to pre-diagnosis levels [43]. Though parallel decreases in spouse physical activity have not been studied among couples coping with cancer, spouse care partners self-report lower physical activity levels than non-caregiving spouses [44]. Marital status and/or spousal physical activity are important determinants of physical activity behavior within the couple [45, 46] and may be explained by the spouse’s support for physical activity in their partner [46, 47]. Having couples exercise together could build the teamwork and support that optimizes the benefits of exercise on the physical and mental health of both patients and their care partners.

The *Exercising Together©* program is a dyadic health management strategy using a teamwork-based approach that adds communication, collaboration, and support between partners during exercise to amplify the benefits of physical training and fortify the relationship. We initially created and piloted *Exercising Together©* in couples who were far past his treatment completion (on average 6 years post-diagnosis). A 6-month long program of *Exercising Together©* showed strong acceptability and led to better physical fitness and signs of improving the relationship [48, 49]. While promising, it is known that the impact of cancer begins at diagnosis and over time can lead to persistent health problems and erode relationships [50, 51]. Additionally, we may have a window of opportunity for behavior change at the initiation of their initial treatment for prostate cancer, as suggested by Demark-Wahnefried [52]. Thus, we may have a better opportunity to prevent long-term adverse outcomes in couples rather than try to reverse health declines and restore relationship health by delivering *Exercising Together©* earlier on in the care trajectory. Radiation therapy may provide a window of opportunity to where exercise can be integrated to mitigate treatment-related effects. By introducing couple-based exercises at the time when physical activity levels typically decline for the patient and his spouse, each partner may avoid inactivity-related declines in health and function and create the support system needed to sustain their physical activity levels after treatment ends. To fit the context of the radiation setting, the length of *Exercising Together©* must shorten and thus a key modification will be to emphasize developing teamwork within the couple and support for one another with a goal that they continue exercising after radiation treatment ends. Thus, we conducted a pilot study to address the following objectives: (1) determine the feasibility and acceptability of an adapted version of *Exercising Together©* for delivery during radiation therapy for prostate cancer and (2) assess preliminary efficacy of the adapted version of *Exercising Together©* on physical, mental, and relational health among prostate cancer patients and their spouse.

## Methods

### Study design and setting

We conducted a single-group, pre-post pilot study of *Exercising Together*© in prostate cancer patients undergoing radiation therapy and their spouses. Oregon Health & Science University (OHSU) Institutional Review Board approved the trial protocol and written informed consent was obtained from participants prior to the baseline study visit. The trial is registered on clinicaltrials.gov (NCT03418025).

### Study sample and recruitment

Men scheduled for radiation therapy for prostate cancer at OHSU and their spouse (or co-residing partner) comprised the target sample. We sought to enroll 10 couples in order to address our feasibility aim whereas effect sizes and confidence intervals on secondary outcomes were calculated to plan future trials. Rolling recruitment occurred directly through provider referral from November 2017 to March 2018 until 10 couples were enrolled. Providers sequentially referred patients who were scheduled to begin radiation therapy and who were married/partnered and residing in the Portland metro area during treatment. Study staff then contacted potentially eligible couples to conduct a full screening for eligibility and obtain informed consent. Eligibility criteria for patients included (1) histologically confirmed prostate cancer; (2) scheduled to receive radiation therapy for prostate cancer; and (3) currently residing with a spouse (or partner) willing to participate. Patients and spouses were eligible if there was (1) no presence of cognitive difficulties that precluded answering survey questions or giving informed consent, (2) no presence of medical conditions, movement or neurological disorders, or medication that contraindicated participation in resistance exercise, and (3) no regular participation in resistance training two or more times per week. Criteria were obtained by self-report or within the electronic medical record. Physician clearance to participate in moderate-intensity resistance training was obtained by the treating radiation oncologist and was also obtained from the spouse’s health care provider if indicated by the American College of Sports Medicine (ACSM) pre-participation screening criteria [53].

### Intervention

*Exercising Together©*, a progressive dyadic resistance training program, was originally designed as a 6-month intervention, but for this trial, we adapted it for delivery over a 5–8-week course of radiation therapy. For the radiation setting, we had to significantly shorten the program so we refocused it to center around couples learning the exercises together and working as a team throughout the routine in order to establish an exercise partnership that could endure after treatment completion. Prior to joining group exercise sessions, each couple completed a 2-h-long orientation session where the trainer taught participants how to do individual exercises and introduced the key concepts and approaches to building teamwork. Participants then attended hourlong group exercise sessions 3 times per week. Exercise sessions were led by two certified ACSM Cancer Exercise trainers and an hour long and included the following elements: (1) dynamic warm-up execises, (2) posture exercises, (3) resistance exercises, and (4) cooldown stretches. The *Exercising Together©* program is grounded in the principles of functional resistance training where exercises focus on muscle groups and movements used in day-to-day activities and included squats, chair stands, lunges, rows, push-ups, bridges, and planks. We also include partnered versions of some exercises where couples performed a movement collaboratively (i.e., plank with hand clap). The resistance training portion of the program progressed from 1 to 3 sets of each exercise and an intensity of 12–15 repetition maximum (RM) to 8–10 RM over their time in the program and based on individual capacity and tolerance. Resistance bands of varying tensions were used to provide overload. We incorporated 5–10 min of postural exercises preceding the resistance exercises to promote proper form and safety among novice exercisers.

The concept of teamwork is woven into all elements of the *Exercising Together©* program and those elements are specifically designed to promote communication, collaboration, and supportive behavior within the couple [54]. One approach used in the program is to have the couple take specific roles during the primary resistance exercises where each partner takes both a *trainer* (i.e., coach) and *exerciser* role. In the *trainer* role, participants become a coach for their partner and assess their capacity for a given exercise, assist them with an exercise by checking form visually or manually, applaud their effort and encourages them, and advise on what they might do for the next set or session (4 A’s). In the *exerciser* role, participants need to receive (i.e., listen) their partner’s feedback and encouragement and respond to it verbally or non-verbally (i.e., nod or high-five) (2 R’s). Partners would perform one set of an exercise in the *trainer* or *exerciser* role and then switch for another set repeating the 4 A’s and 2 R’s of teamwork. To further encourage communication and collaboration within couples, 2–3 additional dyadic versions of exercises were performed (e.g., squats performed face-to-face, lunge with ball pass).

### Procedures

At baseline, participant demographics and health history were collected by self-report. Primary outcomes of feasibility and safety were collected by research staff throughout the trial, while acceptability was assessed with a survey developed by the research team and administered to couples at the end of the intervention. Secondary outcomes of physical, mental, and relationship health on both the patient and the spouse were assessed by both patient-reported and objective measures assessed at baseline and at the end of radiation treatment (5–8 weeks; post-intervention). We readministered surveys at 8-week follow up but performance measures were not collected because at least half of the couples left the vicinity of OHSU after completion of radiation therapy (i.e., couples who permanently reside out-of-state). Since the study was a single-group design, study assessors were unblinded.

### Primary outcomes

#### Feasibility and acceptability

Feasibility was measured by accrual, retention, exercise adherence, and completion of surveys and performance tests. Accrual was calculated as the number of couples who enrolled out of those approached by the referring radiation oncologist. Retention was calculated as the number of couples who remained in the study post-intervention relative to the number enrolled. Exercise adherence was calculated as a percent of the total number of exercise sessions attended by the couple out of sessions prescribed. Completion rates of performance tests and patient-reported outcomes were calculated as a percentage of data collected out of planned collections. Adverse events and/or symptoms related to the study exercise program were assessed on an ongoing basis in classes and recorded by the exercise instructor. Acceptability of *Exercising Together*© by couples was assessed by an evaluation survey completed at the conclusion of the exercise program. The survey had participants rate their overall satisfaction with the program on a 10-point Likert scale from 1 (poor) to 10 (exceptional) as well as their perceptions of the accessibility, effectiveness, and enjoyment of classes on a scale from 1 (strongly disagree) to 4 (strongly agree). Couples were also given the opportunity to provide written open-ended feedback on program content and delivery.

### Secondary outcomes

#### Physical health

##### Physical functioning

The short physical performance battery (sPPB) consists of three timed tests: chair stands, standing balance, and usual gait speed. The sPPB is reliable and sensitive to change [55]. Chair stands time how long it takes participants, in seconds, to rise and sit from a chair five consecutive times as fast as possible. Standing balance includes a sequence of timed stance tests of increasing difficulty. Gait speed (m/s) takes the average of two repeated walks along a 4-m course at a person’s typical pace. Each test is scored 0 (unable) to 4, based on quartiles of performance, and then summed for an overall score of 0 to 12 where higher values indicate higher physical functioning. Since the continuous scores from chair stand and usual gait speed tests are each predictive of poor outcomes in older adults, we also considered these as separate outcomes where shorter times and faster speeds indicate greater lower body strength and better mobility, respectively.

##### Functional capacity

The 400-m walk test is a self-paced, submaximal exercise test and measures cardiorespiratory fitness and functional capacity [56]. Participants completed 10 laps on a 20-m out-and-back course marked by two cones as fast as they could, recorded in minutes. A faster time indicates higher functional capacity.

##### Physical activity

Self-report physical activity was assessed using the four-item Godin Leisure-Time Exercise Questionnaire [57], which measures the average number of self-reported minutes spent participating in general physical activity and moderate-to-vigorous physical activity (MVPA) minutes per week. Participants recall over the past month the average time spent and perceived intensity of recreational exercise for bouts that are > 15 min long. The instrument has a fair correlation to accelerometry for MVPA in cancer patients [58].

#### Mental health

##### Depressive symptoms

The Center for Epidemiological Studies-Depression (CES-D) scale measured the degree of depressive symptoms [59]. Scores range from 0 to 60, with higher scores indicating a greater number of symptoms that occur more often.

##### Anxiety

The Symptom Checklist-90 Anxiety Scale (SCL-90 ANX) [60, 61] which includes 10 items representing anxiety symptoms, scored on a 5 point scale ranging from 0 (not at all) to 4 (extremely), measured anxiety over the previous week. Internal consistency reliability for anxiety is 0.85, with good convergent and discriminant validity [62].

#### Relationship health

##### Dyadic coping

Two types of dyadic coping, active engagement and protective buffering, were measured. *Active engagement* assesses the extent to which the patient and spouse view their partner’s active involvement and joint problem-solving [63, 64]. Participants respond to 5 questions on a 5-item Likert scale from 1 (never) to 5 (very often). Higher scores indicate higher levels of perceived active engagement. The scale has exhibited high Chronbach’s alpha values (0.77 to 0.97) in studies of couples with cancer [63, 65]. *Protective buffering* asks the patient and spouse to assess the extent to which their partner uses hiding concerns and denying worries as protective strategies [63, 64]. Participants respond to 6 items using a Likert scale from 1 (never) to 5 (very often). Higher scores indicate higher levels of perceived protective buffering. The scale has exhibited high Chronbach’s alpha values (0.75 to 0.87) in studies of couples with cancer [63, 65].

##### Strain

Strain is measured with The Role Overload Scale [66], which assesses the extent a partner’s time and energy are exhausted by demands of care for their partner. Higher scores indicate high levels of strain. The scale has exhibited high reliability and construct validity with patient physical function and spouse depression [66–68].

##### Physical intimacy

The Physical Intimacy Behavior scale [69] measures affectional and sexual behavior. Respondents report on a Likert scale from 1 (none of the time) to 4 (most or all of the time) the frequency in which they engage in four affectionate (e.g., touching, kissing, hugging, caressing) and two sexual behaviors (e.g., sexual intercourse, foreplay). Subscales have demonstrated strong internal consistency and construct validity [70].

##### Statistical analysis

Descriptive statistics were completed and reported as means (standard deviations; SD) or frequencies on enrolled couples. Feasibility and acceptability data (enrollment, retention, adherence, and adverse events) were reported as percentages. Effect sizes and 95% confidence intervals (CI) were calculated from baseline to post-intervention and from post-intervention to 8-week follow-up for self-reported outcomes. Effect sizes were reported as those initially suggested by J Cohen [71] and expanded by SS Sawilowsky [72] (0.01: very small; 0.20: small; 0.50: medium; 0.80: large; 1.20: very large; 2.0: huge). Paired *t*-tests were conducted to compare outcomes between baseline and post-radiation and post-radiation and 8-week follow-up. While the alpha level was established at 0.05 for all analyses, the significance of results from analyses should be interpreted cautiously due to the small sample size of a feasibility pilot. Analyses were completed in R version 3.6.1 (R Studio, PBC. Boston, MA, USA).

## Results

### Participant characteristics

Characteristics of enrolled patients (*n* = 10) and spouses (*n* = 10) are reported in Table 1. Average age was 71.6 ± 7.9 and 69.4 ± 5.4 years for patients and spouses, respectively, and couples had been together for a mean duration of 38.3 years. All participants were non-Hispanic white while the majority had at least a high school education. All participants were obese based on self-reported body mass index (BMI, calculated as kg/m^2^). Half of enrolled couples were Oregon residents and the other half were temporarily residing near OHSU for radiation therapy and thus during the study intervention. Patients were on average 22.7 ± 29.8 months from their initial prostate cancer diagnosis, 40% were on androgen deprivation therapy (ADT) and 12.5% reported metastatic disease. Length of prescribed radiation therapy ranged from 5.5 to 7 weeks, with a median of 6.3 weeks.

**Table 1:** Demographic characteristics of couples

Characteristic	Patient (***n*** = 10)	Spouse (***n*** = 10)
Age (years) [mean (SD)]	71.6 (7.9)	69.4 (5.4)
Sex		
% Male	100%	
% Female		100%
Race (% white)	100%	90%^a^
Ethnicity (% Non-Hispanic)	100%	100%
Education (% above high school)	75%	75%
Employment status		
% Retired	70%	80%
% Working full or part-time	30%	20%
Oregon resident (%)	50%	50%
Marital status (% married)	100%	100%
Length of relationship (years) [mean (SD)]	38.1 (16.8)	38.4 (17.22)
BMI (kg/m^2^) [mean (SD)]	33.7 (6.4)	31.2 (9.7)
***Patient characteristics***		
Time since diagnosis (months) [mean (SD)]	22.7 (29.8)	NA
Currently on ADT (%)	40%	NA
Metastatic disease (%)	12.5%^b^	NA

Abbreviations: *BMI* body mass index, *ADT* androgen deprivation therapy, *NA* not applicable

^a^Missing data *n* = 1

^b^Missing data *n* = 3

### Feasibility and acceptability

**Fig. 1:**
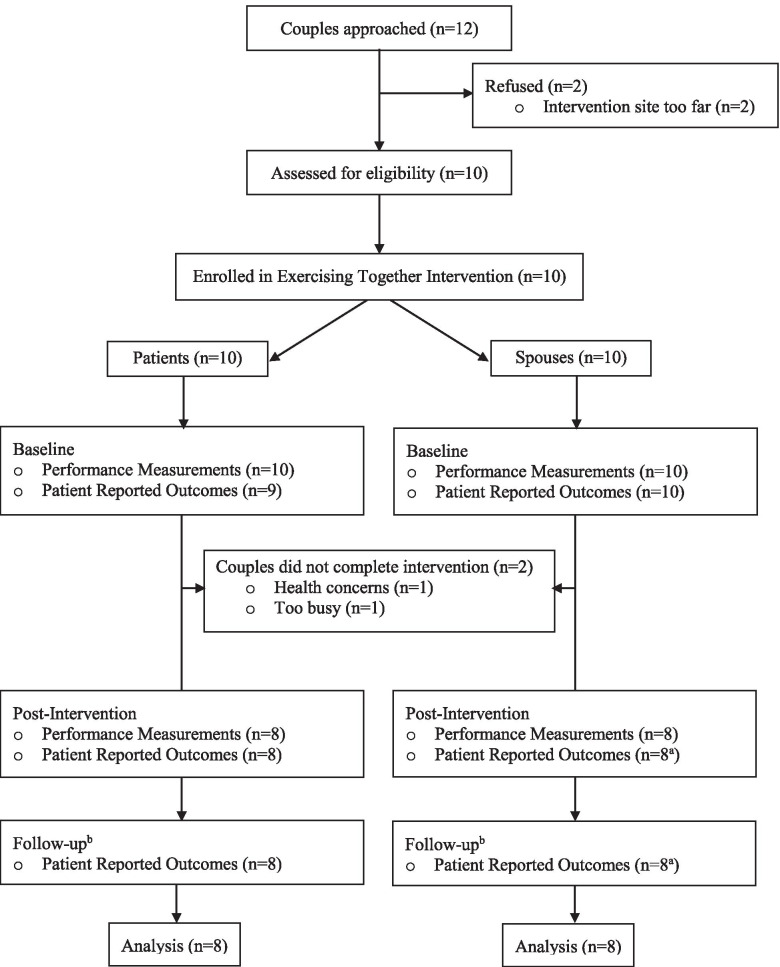
Participant flow throughout *Exercising Together©* during radiation therapy

The *Exercising Together©* intervention was delivered between November 2017 and May 2018. Figure 1 provides details of participant flow through the study. The treating oncologist referred 12 couples who were eligible and expressed interest in participating in the study. Two couples declined to participate because of the length of their commute and ten couples were deemed eligible and enrolled, translating to an 83% accrual rate. Two couples discontinued the exercise program after the first week of participating due to a new health problem (*n* = 1 patient) and concerns over the study commitment (*n* = 1 couple) translating to an 80% retention rate. Data on couples that did not complete the intervention were excluded from outcome analyses. Average couple adherence to planned sessions was 83% (95% CI: 73–93%) with only four sessions where an individual spouse did not attend with her husband. Completion rates of performance testing and surveys at post-intervention and follow-up was 100% for couples, with the exception of the Physical Intimacy Behavior survey, where 1 spouse declined to complete some answers on the affectionate behavior subscale and 2 spouses declined to complete answers on the sexual behavior subscale, either post-intervention or follow-up. No adverse events related to the study exercise program were reported.

Couples rated the class highly (mean scores on a 1–10 scale: patients = 9.4 ± 1.9 and spouses = 10.0 ± 0.0) and 88% of patients and 75% of spouses stated that they would prefer a couple-based group class over a general community fitness class. Overall, the classes received positive feedback regarding access, instructors, motivation, and content (Table 2). Patient and spouse responses to open-ended questions about program satisfaction are in Table 3. Frequent themes reported included collaboration, motivation, support, and sense of being more capable and strong. Couples felt empowered by the classes and that they were united in facing the cancer diagnosis and treatment together. Many reported the classes being beneficial and a memorable experience with intentions to continue the exercises beyond the intervention period.

**Table 2:** Patient and spouse perceptions of *Exercising Together©* during radiation therapy at post-intervention

Perception	Patient (*n* = 8)	Spouse (*n* = 8)
	Mean (SD)	Mean (SD)
*Accessibility*		
Classes were easy to get to	3.6 (0.8)	3.6 (0.5)
Felt safe in class	4.0 (0.0)	4.0 (0.0)
Free classes were a large incentive	3.5 (0.8)	3.9 (0.4)
Instructors were professional and credible	3.9 (0.4)	4.0 (0.3)
Instructors adapted/modified exercises	3.9 (0.4)	4.0 (0.0)
*Effectiveness*		
Instructors were knowledgeable about cancer	3.3 (0.8)	3.9 (0.4)
Instructors were a good role model	3.9 (0.4)	4.0 (0.0)
Instructors were encouraging	4.0 (0.0)	4.0 (0.0)
Classes helped motivation to exercise	3.9 (0.4)	4.0 (0.0)
Felt exercises improved my health and fitness	3.8 (0.5)	3.9 (0.4)
*Enjoyment*		
Enjoyed the type of exercise done in class	3.9 (0.4)	4.0 (0.0)
Enjoyed exercising with spouse/partner	3.9 (0.4)	4.0 (0.0)

Scale: 1: strongly disagree; 2: disagree; 3: agree; 4: strongly agree

**Table 3:** Patient and spouse comments on acceptability of *Exercising Together©* during patient’s radiation therapy

**Patient comments**	
• I liked that it was a couple’s class. The experience was pleasant and beneficial. It exceeded my expectations. The biggest thing is it encouraged and supported communication between us focusing and working toward common goal. I think “communication” can certainly suffer during stressful times. I/we certainly weren't prepared for cancer.	
• Very good idea having this kind of class, I feel very lucky to have been a part of it. This is a very good way to deal with the cancer problem. I hope you continue this program, I felt it was a great benefit to everyone.	
• Motivated me to stay active. Was good for spouse to get some exercise. Motivation to be positive, meet role models and share cancer experience. Was good to see my spouse be involved.	
• A unique program as most fitness programs are only for individual, not couples. I don’t know any fitness programs that enable couples to learn how to coach each other. Highly recommend the partner exercise program as it enables empathy, understanding, respect, sharing, caring that are essential to marriage. I will never forget the experience.	
• Very good program, really helped get my spouse to be more involved in exercise...more capable than she would ever have thought. A FUN diversion to the daily radiation treatments.	
• I enjoyed exercising with my wife. Exercising together had much more value than if I did it by myself. I always looked forward to each class. My treatments are over and we plan on attending these exercise classes as long as we can.	
• Very good idea to do couples, especially for the motivation. The motivational value as well as the coaching when we do the exercises as a couple at home.	
• The best part about the program was learning to help each other to do the exercise correctly.	
**Spouse comments**	
• The classes were really perfect for us, it felt empowering and we really grew closer because of it. My spouse is a work-a-holic, so not much interaction. We had the opportunity to “work” together. Gives purpose, like we are fighting the cancer with all we had.	
• Really did help me keep moving. I would have just gone home and sat if it wasn’t for the class. I most definitely would recommend this class for everyone going through cancer treatment and their partner because it brings couples closer together. You work as one unit. You help each other and strengthen each other and learn to support each other in ways you don't necessarily think of otherwise.	
• It was fun and something we had never done, but I think we will continue to do together.	
• Liked the social and communication between the couples. Formed new friendships. This class provides a connection and informal group support for radiation. Keep this program of exercise with couples.	
• I know this was a boost for my husband’s moral through his treatment process.	
• Being with my spouse put me in a safe, non-judgmental, supportive place right off the bat. It was the bright spot in each day of treatment it fell on, and the strength we gained felt SO good. I do believe the fatigue was lessened for my spouse. We both hope this gets put into the treatment protocols. We gained a great deal from being in the classes.	
• The partnered exercise program is a great idea and we are hoping that we will be able to carry it on and keep up what we have learned and gained. It's always good to exercise with a partner. It helped to keep our spirits up and our bodies energized to the extent that one can be during radiation.	
• I do not think my husband would have participated if they were not for couples. They seemed to brighten the day to spend some time with other couples going through the same processes.	

### Physical health

Physical health outcomes improved in both patients and spouses across the intervention period (Table 4). Among patients post-intervention, there was a significant improvement and large effect from the intervention on gait speed (mean difference (MD) = 0.1; 95%CI: 0.1, 0.2; *p* = 0.003; *d* = 0.94), and a significant improvement with a small effect size on functional capacity (MD = −0.6; 95%CI: −0.9, 0.3; *p* = 0.002; *d* = −0.42). Nearly significant improvements in sPPB and chair stand time also occurred in patients, with medium effect sizes. Among spouses post-intervention, there was a significant medium and large effect size from the intervention on sPPB and chair stand time (MD = 1.3; 95%CI: 0.3, 2.2; *p* = 0.02; *d* = 0.71 and MD = −4.9; 95%CI: −8.0, 1.63; *p* = 0.009; *d* = −0.87, respectively). Physical health measurements were not collected at the 8-week follow-up.

**Table 4:** Physical, mental and relationship health outcomes for patients and spouses completing *Exercising Together©* during radiation therapy

Characteristic	Patient (***n*** = 8)							Spouse (***n*** = 8)						
	Baseline (T1)	Post-Intervention (T2)	Follow-Up (T3)	Change (T1–T2)	Change (T2–T3)	*p*-value*	Cohen’s d*	Baseline (T1)	Post-intervention (T2)	Follow- UP (T3)	Change (T1–T2)	Change (T2–T3)	*p*-value*	Cohen’s d*
	Mean (SD)	Mean (SD)	Mean (SD)	Mean (95%CI)	Mean (95%CI)			Mean (SD)	Mean (SD)	Mean (SD)	Mean (95%CI)	Mean (95%CI)		
**Physical health**														
5-time sit-to-stand (s)	13.9 (4.6)	11.2 (2.6)	–	− 2.8 (− 5.7, 0.2)	–	0.06/–	− 0.60/–	15.7 (5.5)	10.9 (2.3)	–	− 4.9 (− 8.0, − 1.63)	–	0.009/–	− 0.87/–
Gait speed (m/s)	0.9 (0.2)	1.1 (0.1)	–	0.1 (0.1, 0.2)	–	0.003/–	0.94/–	1.0 (0.1)	1.1 (0.3)	–	0.1 (− 0.1, 0.2)	–	0.20/–	0.64/–
sPPB	10.5 (1.5)	11.4 (0.7)	–	0.9 (− 0.2, 1.9)	–	0.09/–	0.58/–	10.0 (1.8)	11.3 (1.4)	–	1.3 (0.3, 2.2)	–	0.02/–	0.71/–
Time to walk 400 m (min)	5.1 (1.4)	4.5(1.3)	–	− 0.6 (− 0.9, − 0.3)	–	0.002/–	− 0.42/–	5.2 (1.8)	5.1 (2.1)	–	− 0.1 (− 0.5, 0.3)	–	0.61/–	− 0.05/–
**Mental health**														
Depressive symptoms	12.8 (12.7)	12.3 (11.7)	9.0 (11.0)	− 0.5 (− 5.9, 4.9)	− 3.25 (− 7.7, 1.2)	0.83/0.13	− 0.04/− 0.26	14.5 (11.6)	9.4 (9.7)	7.5 (7.3)	− 5.1 (− 11.8, 1.6)	− 2.0 (− 5.8, 1.8)	0.11/0.25	− 0.44/− 0.17
Anxiety	4.4 (5.3)	2.1 (4.1)	3.6 (7.2)	− 2.3 (− 3.8, − 0.7)	1.5 (− 1.1, 4.1)	0.01/0.22	− 0.43/0.28	3.0 (2.8)	1.5 (1.7)	0.8 (0.9)	− 1.5 (− 3.3, 0.3)	− 0.8 (− 2.4, 0.9)	0.09/0.32	− 0.53/− 0.27
**Relationship health**														
Active engagement	21.8 (2.5)	24.3 (0.9)	21.9 (3.2)	2.5 (0.7, 4.3)	− 2.4 (− 5.2, 0.5)	0.01/0.09	0.98/− 0.93	18.8 (4.6)	19.0 (4.8)	17.4 (2.9)	0.25 (− 2.2, 2.7)	− 1.6 (− 5.4, 2.1)	0.82/0.34	0.05/− 0.36
Protective buffering	12.1 (2.5)	10.5 (3.1)	9.9 (2.3)	− 1.6 (− 5.1, 1.9)	− 0.6 (− 2.3, 1.0)	0.31/0.41	− 0.64/− 0.25	14.1 (4.1)	14.0 (4.8)	11.1 (3.1)	− 0.1 (− 2.9, 2.6)	− 2.9 (− 7.9, 2.1)	0.92/0.22	− 0.03/− 0.70
Strain	2.9 (2.0)	3.1 (1.9)	2.7 (2.1)	0.3 (− 0.9, 1.4)	− 0.5 (− 1.9, 0.9)	0.63/0.43	0.12/− 0.25	2.9 (2.2)	3.3 (2.9)	2.4 (0.9)	0.4 (− 2.3, 2.0)	− 0.9 (− 3.0, 1.2)	0.75/0.36	0.17/− 0.39
Affectionate behavior	11.3 (3.4)	11.9 (1.8)	11.0 (2.6)	0.63 (− 2.0, 3.3)	− 0.9 (− 2.3, 0.6)	0.59/0.19	0.19/− 0.26	13.6 (3.3)	12.1^a^ (3.0)	12.7^a^ (4.2)	− 1.6 (− 3.9, 0.6)	0.6 (− 2.1, 3.3)	0.13/0.62	− 0.50/0.18
Sexual behavior	2.6 (0.9)	2.4 (1.1)	2.8 (1.2)	− 0.3(− 1.0, 0.5)	0.4 (− 0.2, 1.0)	0.45/0.20	− 0.27/0.41	2.5 (1.2)	2.7^b^ (1.2)	3.2^b^ (2.4)	0.2 (− 0.3, 0.6)	0.5 (− 0.8, 1.8)	0.36/0.36	0.14/0.41
**Physical activity**														
Total (min/week)	175.6 (140.0)	280.6 (206.2)	253.8 (262.8)	105.0 (− 29.6, 239.6)	− 26.9 (− 223.0, 169.2)	0.11/0.76	0.75/− 0.19	158.4 (132.6)	338.0 (205.4)	198.1 (192.8)	179.6 (55.4, 303.7)	− 139.9 (− 266.5, − 13.3)	0.01/0.03	1.35/− 1.06
Moderate-to-vigorous (min/week)	51.9 (96.3)	78.8 (156.2)	86.3 (100.7)	26.9 (− 31.8, 85.5)	7.5 (− 78.1, 93.1)	0.31/0.84	0.28/0.08	87.2 (77.1)	103.8 (105.4)	90.6 (101.2)	16.6 (− 59.1, 92.2)	− 13.1 (− 85.9, 59.7)	0.62/0.68	0.21/− 0.17

Abbreviations: *T* time point, *SD* standard deviation, *sPPB* short physical performance battery, *–* data not collected

*p-values and Cohen’s *d* effect size depicted for (T1–T2) and (T2–T3) using SD from baseline

^a^Missing data *n* = 1, declined to answer

^b^Missing data *n* = 2, declined to answer

### Mental health

Depressive symptoms in patients and spouses decreased across all time points (Table 4). Patient depressive symptoms decreased post-intervention with decreases sustained at follow-up, but changes were not significant and yielded very small to small effect sizes. Similar trends were observed in spouses. Anxiety decreased significantly in patients post-intervention (MD = −2.3; 95%CI: −3.8, 0.7; *p* = 0.01; *d* = −0.43) and increased slightly at follow up, with small effect size across both time periods. Anxiety decreased among spouses post-intervention and follow-up but not significantly so and with medium and small effect sizes.

### Relationship health

Patterns of change for relationship variables were similar for patients and spouses across all time points (Table 4). There was a significant increase and large effect size for active engagement at post-intervention among patients (MD = 2.5; 95%CI: 0.7, 4.3; *p* = 0.01; *d* = 0.98) that decreased at follow-up to levels similar to baseline. Among spouses, changes in active engagement were non-significant and very small or small effect sizes at both time points. For the remaining outcomes (protective buffering, strain, affectionate behavior, and sexual behavior) there was no significant change at post-intervention or follow-up and effect sizes were small to medium for both patients and spouses.

### Physical activity

Changes in total physical activity and MVPA were similar for patients and spouses (Table 4). Total physical activity non-significantly increased in patients post-intervention but decreased at follow-up with a medium effect size. Total physical activity among spouses significantly increased at post-intervention, but decreased at follow-up with very large to large effect sizes across time periods, respectively (MD = 179.6; 95%CI: 55.4, 303.7; *p* = 0.01; *d* = 1.35 and MD = −139.9; 95%CI: −266.5,13.3; *p* = 0.03; *d* = 1.06). Patients and spouses reported non-significant increases in MVPA post-intervention that continued to increase at follow-up in patients but decline in spouses. All effect sizes for MVPA were small.

## Discussion

Our adaptation of *Exercising Together©* is the first time a dyad-focused approach using a partnered exercise program has been implemented and evaluated during cancer treatment. We assessed the feasibility of implementing *Exercising Together©* during radiation treatment in order to combat the triple threat of a cancer diagnosis on patient and spouse physical, mental, and relationship health early on in the treatment trajectory when couples are seeking resources and strategies to cope with a new illness. The program was found to be both feasible and acceptable, demonstrated by successful enrollment, retention, and adherence rates that met or exceeded target thresholds, along with positive qualitative feedback from participants. In the shorter period that couples participated in *Exercising Together©*, both patients and spouses experienced improvements in physical functioning and reductions in anxiety and depressive symptoms, while relationship outcomes either remained steady or improved. Couples also became more generally physically active at a time when patients and spouses struggle to do so. After 8 weeks of follow-up, some of the self-reported improvements during training began to dissipate though many remained higher than pre-treatment levels.

The first study of *Exercising Together©* showed strong acceptability and improved physical, mental, and relationship outcomes in couples coping with prostate cancer compared to usual care controls [48, 49, 73]. While promising, the original program was six months long and enrolled couples long after men completed primary treatment, thus it was not known whether or not substantially shortening the length of the program so that it could be administered during radiation therapy would alter the feasibility and efficacy of this dyadic approach. Based on qualitative feedback, couples who completed the program indicated high acceptability based on extremely positive ratings overall and in specific areas of accessibility, effectiveness, and enjoyment. Accrual, retention, and adherence rates were all above 80% pointing to high feasibility of introducing an exercise program directed at both patients and spouses when much of their time and effort is planned around daily radiation treatments. However, we felt this moment in time was also a window of opportunity for behavior change [52] and, if sessions coincided with radiation treatments for a single visit to the cancer center, would reduce a barrier of time and travel to a separate exercise facility [74]. This was particularly convenient for couples residing within the Portland metropolitan area so they could avoid extra travel for exercise, but also for couples from out of state who temporarily relocated to be nearby the cancer center. Despite these conveniences, couples who refused to participate and one couple who dropped from the intervention cited reasons of time and travel. Even in the radiation setting the program was delivered in a group format thus it is likely, as well as evident from participant comments, that social support probably contributed to strong retention and adherence rates. Previous studies of group exercise in patients undergoing ADT and radiation therapy have reported slightly lower adherence rates (69–79%) [75, 76], though, suggesting that the involvement of the spouse specifically may boost adherence to supervised training.

Our preliminary data suggests that *Exercising Together©* implemented over a course of radiation therapy led to improvements in physical and mental wellbeing during a time that patients and spouses typically experience declines in physical function and worsening mood [17–22]. Across all objective measures of physical functioning, patients and spouses improved with several changes yielding medium to large effect sizes. These findings are consistent with our prior study of *Exercising Together©* delivered for six months post-treatment [49], but also aligned with two prior studies of aerobic or resistance exercise during radiation therapy for prostate cancer [77, 78] and with newer exercise guidelines for cancer survivors that suggest that 30 min of resistance and/or aerobic exercise done 3 times per week can improve physical functioning within 8–12 weeks [39]. Physical functioning also improved in spouses which is a novel finding since prior exercise studies have either not included spouses or did not measure outcomes in spouse participants [79, 80]. Both patients and spouses had low scores on all tests of physical function at baseline suggesting that couples may already be at risk of mobility disability before enduring more cancer treatment [81], but also that exercise may be particularly effective and helpful in persons with low initial functioning [82]. Even with this lower capacity, couples were able to fully participate in the program as evidenced by an absence of program modifications and adverse events.

Similar to observations for outcomes of physical health, depressive symptoms and anxiety lessened in both patients and spouses throughout radiation treatment. Both partners in a couple often experience high levels of psychological distress from the cancer experience [17–22], and patients and spouses may be at an even higher risk for anxiety than for depression [24]. Relatedly, we observed a greater impact of partnered exercise on anxiety than depressive symptoms that reached significance for patients. Effect sizes for mental health outcomes were not as large as those for physical health outcomes, which might align with observations from the ACSM exercise guidelines which found stronger evidence for psychosocial benefits of aerobic training alone or in combination with resistance training over resistance training alone [39]. Though we found evidence for a benefit of resistance training on anxiety in patients, it is possible that adding aerobic exercise could lead to broader improvements. Even after the program ended depressive symptoms in both partners and anxiety in spouses continued to decline, though anxiety tended to increase in patients. These changes could suggest that some elements of the program may have persisted after formal training stopped and contributed to lessened symptoms; however, perhaps continuing a structured exercise program could prevent rising anxiety after treatment completion.

Changes in several measures of relationship health were less consistent with those observed for individual physical and mental health outcomes. Dyadic coping constructs tended to improve in patients throughout the program reaching significance for active engagement but changes reversed or slowed at follow-up, while changes among spouses were relatively flat for both constructs throughout both time periods. Strain rose and fell in patients and spouses across the intervention and follow-up periods, coincident with treatment and recovery. Both constructs of physical intimacy remained relatively unchanged over intervention and follow-up. In our prior study of a longer, post-treatment program of *Exercising Together©*, spouses reported higher levels of affectionate behavior toward their husbands than spouses in a control group. Yet, during radiation therapy, men tended to be more responsive to the adapted intervention than spouses in measures of dyadic coping. There is not much known about whether and how relationship outcomes typically change during and after radiation therapy without any intervention, and without a control group in this pilot, one could only speculate about whether or not participation in partnered exercises stabilized coping behaviors and intimacy or had little effect on them.

From qualitative feedback, both patients and spouses commented that the program provided support for exercise and opportunities for them to work together by collaborating and communicating with one another. These constructs of support, collaboration, and communication are elements of effective dyadic health management strategies that we have intentionally embedded into *Exercising Together©*, and in the shorter version of the program, we increased the emphasis on teamwork to reinforce these constructs so they could persist past treatment. Indeed, some couples experience marital distress well after prostate cancer treatment is completed [83] so the durability of dyadic interventions should be systematically evaluated.

One reason for increasing the emphasis on teamwork in the shorter version of *Exercising Together©* was to promote shared engagement in exercise beyond the intervention period. Physical activity levels are known to decline during cancer treatment and rarely return to pre-treatment levels later on [43], while care partners tend to have high rates of physical inactivity [44]. Total physical activity and MVPA increased over the course of the intervention for both patients and spouses with very high effect sizes for total physical activity. MVPA continued to increase over follow-up in patients whereas total physical activity started to drop within the couple. MVPA and sedentary time are closely linked in married partners [84] and social support provided by a spouse predicts higher MVPA [85]. Though not specific to cancer, a recent meta-analysis found that dyadic interventions with shared target-oriented goals had larger effect sizes on physical activity change compared to interventions where only the individual was targeted [86]. While spousal involvement in interventions during chronic illness is connected with long-term maintenance of health behavior change [87], and this may be the case for patients in our pilot, whether and how long this can be maintained in patients and spouses remains to be determined.

Our study had notable strengths, including our novel dyadic health management strategy tested for the first time at the initial treatment of their prostate cancer with radiation therapy, our focus on dyadic health outcomes, and planning the intervention around treatment to reduce barriers to participation. By initiating exercise at the start of radiation therapy declines associated with the treatment experience could be prevented or lessened, making the program appropriate for all couples regardless of health status. Our study also has an obvious limitation with a single group, pre-post test design that precludes us from drawing strong conclusions from our data in the absence of a control group. It is possible that within this uncontrolled trial outcomes naturally improved. Our sample was small and limited to patients referred by the treating radiation oncologist, thus we are unsure of the potential reach of this program to couples treated at other cancer centers or of the characteristics of couples not referred to the study. For example, patients and partners in our study were, on average, obese; thus, it is possible that our provider preferentially referred couples he thought might benefit the most from participation. We were also unable to assess performance-based measures at 8-weeks follow-up since half of the couples left the vicinity of the study site and could not return for further testing. If future studies wish to assess the durability of exercise-related changes in objectively assessed physical functioning after radiation treatment this may only be possible in a subgroup or other methods to obtain measures at a distance, possibly through video-conference technology [88], might need to be considered.

## Conclusion

Dyadic interventions that simultaneously address the physical and emotional needs of the cancer patient, spouse, and their relationship could have broader health impacts than interventions aimed at the patient alone. A shorter version of our *Exercising Together©* program adapted for delivery during radiation treatment for prostate cancer was feasible and highly acceptable in patients and spouses. The dyadic resistance training intervention showed preliminary evidence that it can improve physical, mental, and relational health during a period in the cancer trajectory when dyadic health tends to decline. A larger controlled trial to determine the efficacy and durability of participating in *Exercising Together©* during radiation treatment for prostate cancer on dyadic health is warranted. The delivery of this program in the context of a cancer center was an important feature that both facilitated participation by provider referral and reduced barriers to participation around time and travel constraints. Thus, a future trial should also investigate implementation strategies, possibly using a hybrid efficacy-implementation trial design, to speed translation to clinical practice.

## Data Availability

Data are available upon request.

## Abbreviations

OHSU: Oregon Health and Science University
ACSM: American College of Sports Medicine
RM: Repetition maximum
sPPB: Short physical performance battery
MD: Mean difference
BMI: Body mass index
MVPA: Moderate-to-vigorous physical activity
ADT: Androgen deprivation therapy
